# How Visual Body Perception Influences Somatosensory Plasticity

**DOI:** 10.1155/2018/7909684

**Published:** 2018-03-11

**Authors:** Esther Kuehn, Burkhard Pleger

**Affiliations:** ^1^Aging and Cognition Research Group, DZNE, 39120 Magdeburg, Germany; ^2^Center for Behavioral Brain Sciences Magdeburg, 39120 Magdeburg, Germany; ^3^Department of Neurology, BG University Hospital Bergmannsheil, Ruhr-University Bochum, 44789 Bochum, Germany

## Abstract

The study of somatosensory plasticity offers unique insights into the neuronal mechanisms that underlie human adaptive and maladaptive plasticity. So far, little attention has been paid on the specific influence of visual body perception on somatosensory plasticity and learning in humans. Here, we review evidence on how visual body perception induces changes in the functional architecture of the somatosensory system and discuss the specific influence the social environment has on tactile plasticity and learning. We focus on studies that have been published in the areas of human cognitive and clinical neuroscience and refer to animal studies when appropriate. We discuss the therapeutic potential of socially mediated modulations of somatosensory plasticity and introduce specific paradigms to induce plastic changes under controlled conditions. This review offers a contribution to understanding the complex interactions between social perception and somatosensory learning by focusing on a novel research field: socially mediated sensory plasticity.

## 1. Introduction

The tactile modality is the first to develop in a human embryo and has important implications for human sensation, action, and cognition. This review addresses the specific question how social cues influence the functional architecture and plasticity of the human somatosensory system. Social neuroscience is a rapidly developing field, but the specific influence of social cues on somatosensory perception is still an underinvestigated topic. Here, we first introduce basic mechanisms of tactile plasticity and learning, such as Hebbian plasticity, GABAergic learning mechanisms, and deprivation-related plasticity (Section 2). Then, we discuss the influence of social cues on human somatosensory cortex functioning and synthesize evidence on the neuronal pathways and experimental conditions that induce nonafferent (visually driven) activity in the human somatosensory system (Section 3). Before combining both research streams to answer the final question (“How do socially-induced ‘resonance' responses in the somatosensory system influence tactile plasticity?”), we provide an overview over the role of touch in human cognition to broaden the scope in which the results can be discussed (Section 4). Finally, we use the introduced frameworks (tactile plasticity, Section 2; socially induced “resonance” responses in the somatosensory system, Section 3; and the role of touch in human cognition, Section 4) to discuss the influence of social cues on tactile plasticity and learning at multiple levels (both mechanistic and cognitive) and its consequences for human behavior in healthy participants, and in patients (Section 5). Whereas the first three sections therefore provide relevant background information, the final section combines the introduced research streams to focus on socially mediated tactile plasticity. We focus on the literature offered by human cognitive and clinical neuroscience, while sometimes referring to animal studies when specific plasticity mechanisms are introduced. This review offers a contribution towards the development of a better understanding of the complex interactions between social perception and somatosensory learning by focusing on a novel and rapidly developing research field: socially mediated sensory plasticity.

## 2. Plasticity Mechanisms in the Somatosensory System

Perceptual learning is the specific modification of perception following sensory experience. This, in turn, involves structural and functional changes in primary sensory cortices [1]. In tactile learning, most of our knowledge about brain plasticity is derived from primary somatosensory cortex (S-I). Larger representations of certain body parts, such as the fingers or lips, are partly due to higher receptor densities reflecting higher demands for cortical processing. These cortical body maps in animals and humans, however, are dynamic constructs that are constantly remodeled by changes in the sensory input statistics throughout life. Structural myelin borders between major body part representations such as the hand and the face in human S-I [2, 3] may to a certain extent limit such plastic changes [4]. Despite the traditional view that perceptual learning requires attention or reinforcement, there is also evidence that only the timing of input statistics can mediate cortical plasticity [5–11]. In fact, since Hebb [12] and even since James [13], the aspect of simultaneity has become a metaphor in neuronal plasticity.

An important feature of the Hebbian metaphor is the coincident pre- and postsynaptic firing of synapses that evokes long-lasting changes in synaptic efficacy. First evidences that temporally correlated activity is required for input-dependent modification in synapses come from the hippocampus in rats [14] and Aplysia ganglia [15]. Although pairing of synaptic inputs and outputs has been hypothesized to play a key role in mediating plastic changes [16–18], more recent evidences suggest that Hebbian plasticity also occurs at dendritic spines without simultaneous pre- and postsynaptic activation [19].

In vitro and computational studies suggested that beyond a simultaneous activation of pre- and postsynaptic cells, there is a “critical time window” of spiking for synaptic modification that is highly specific to certain brain regions [20–26]. These activity-induced changes can occur in vitro at a precision of down to a few milliseconds thus influencing the strength and sign of synaptic plasticity. A critical window for the induction of long-term potentiation (LTP) and long-term depression (LTD) has been characterized in rat hippocampal neurons. This window is about 40 ms long and is temporally asymmetric. Bi and Poo found that repetitive postsynaptic spiking that precedes presynaptic activation in a time window of 20 ms (60 pulses at 1 Hz) resulted in LTP, whereas postsynaptic spiking 20 ms before repetitive presynaptic activation led to LTD. Apart from a critical window for modification of synaptic excitability, synaptic strength and specific postsynaptic cell types (NMDA and GABAergic receptors) are crucial factors for the induction of LTP and LTD [21]. In line with these findings, an almost identical time dependence was described in developing *Xenopus* retinotectal synapses [27]. In contrast, neurons in cortical layer 4 of somatosensory cortex seem to have only a symmetric time window for LTD within ±10 ms, whereas no long-term potentiation in synaptic response was observed [28].

Also, the deprivation of sensory input leads to changes in the functional architecture of S-I representations. In limb amputees, where afferent input to the limb is absent, the cortical representation of the face shifts towards the territory of the hand [29, 30]. Recent findings indicate, however, that these shifts are smaller than originally suggested and that the representation of the absent hand is still preserved [30–32]. Also, altered hand use in amputees induces somatomotor plasticity in amputees. Makin et al. [33] showed that deprived sensorimotor cortex is employed by whichever limb individuals are overusing.

Perceptual learning occurs continuously throughout life and involves either transient or persistent changes in central nervous perceptual systems, which in turn improves the ability to respond to the environment [34–36]. To obtain information about the role of input statistics alone in mediating plasticity in perceptual systems, several protocols, in which neuronal activity was generated by associative pairing, have been developed [37]. In adult rats, for instance, it has been shown that “whisker pairing,” which involves trimming of whiskers except two neighboring vibrissae, resulted in changes in sensory neural activity [37].

Based on the same idea of paired sensory inputs, several studies in animals and humans demonstrate that a variation of input statistics using passive stimulation protocols results in cortical plasticity [5–11]. Godde and coworkers developed a stimulation protocol, called “tactile coactivation,” to receptive fields on the hindpaw of adult rats. The basic idea behind this stimulation protocol was to coactivate a large number of receptive fields in a Hebbian manner in order to strengthen their mutual interconnectedness. Coactivation consists of tactile stimuli that were presented at different interstimulus intervals from 100 to 3000 ms in pseudorandomized order with a mean stimulation frequency of 1 Hz. Coactivation of the hindpaw for 3 hours revealed a selective enlargement of corresponding cortical maps and receptive fields [38]. To investigate the perceptual relevance of the coactivation effect, tactile spatial discrimination performance was tested in humans. Coactivation of receptive field on the fingertip resulted in an improved tactile spatial discrimination ability that lasted for 24 hours. Perceptual changes were highly selective because no transfer of improved performance to nonstimulated fingers was found [5].

Pleger et al. studied the relation between those coactivation-induced perceptual changes and parallel plasticity in human S-I. Using somatosensory-evoked potential (SSEP) mapping [6] and functional magnetic resonance imaging (fMRI) [7], they found that coactivation-induced changes in tactile acuity were reflected in the degree of cortical reorganization. The cortical representation of the coactivated finger in S-I post- versus precoactivation was considerably larger on the coactivated side than on the control side [6, 7]. Using fMRI, Pleger et al. extended the focus on cortical plasticity in the secondary somatosensory cortex (S-II). Contralateral to the coactivated finger, S-II presented with enhanced BOLD signal change comparable to the effects observed in S-I. In line with previous findings [5], tactile discrimination thresholds recovered to baseline 24 hours after coactivation. Furthermore, the relation between cortical plasticity in S-I and perceptual changes was linearly correlated, indicating a close link between the magnitude of plastic changes and coactivation-induced spatial discrimination improvements [6, 7]. In S-II, no such brain-behavior relationship was observed, which might be due to the less fine-grained representational organization of S-II as compared to S-I [39]. Coactivation-induced cortical plasticity together with perceptual improvements was found not only in the young brain but also in older adults suggesting that coactivation-induced effects occur continuously throughout life [40].

To shed light on the underlying cellular mechanisms mediating this specific type of perceptual learning and associated cortical plasticity, Dinse et al. manipulated coactivation-induced perceptual learning with different drugs that specifically block or stimulate central nervous receptors assumed to play a key role in mediating brain plasticity [8, 9]. Under memantine, a NMDA receptor blocker [41], they found that both perceptual improvements and associated cortical plasticity were blocked [8]. Under a single dose of amphetamine, which is known to modify long-term changes in synaptic function [42], perceptual improvements and cortical plasticity were boosted [8]. These results emphasize the prominent role of the NMDA receptor in mediating coactivation-induced perceptual improvements and cortical plasticity. Monoaminergic substances such as amphetamine instead seem to facilitate this specific type of perceptual learning. In line with these experimental findings, perceptual learning after the application of coactivation was shown to be dependent on GABAergic mechanisms. Tactile discrimination improvement was completely abolished by lorazepam, indicating that this GABA_A_ receptor agonist acts to suppress the coactivation-induced effect [9]. Positive correlations between levels of GABA in primary brain regions and sensory discriminative abilities stress the importance of GABA in increasing the perceived contrast of sensory percepts [43].

## 3. The Influence of Social Cues on the Somatosensory System

A dominant theory holds that similar motor areas in the brain are activated when a specific action is either observed or executed [44–46]. This reactivation during observation is often referred to as “neuronal resonance” response [47] and has been investigated quite extensively in recent years [48–51]. Neuronal resonance responses in the motor system are supposed to allow an understanding of others' goals, intentions, and motor plans [44, 46, 52–55] and can lead to interference effects between one's own actions and observed actions [55]. The concept of neuronal resonance was subsequently transferred to other domains, such as the domain of pain, emotion, and touch [52, 56–60], but also to the domain of touch [60–65]. Neuronal resonance responses in the pain matrix or sensory cortices are assumed to trigger shared affective or sensory states, respectively, between observed person and observer.

S-I holds dense connections to S-II, the parvocellular area (PV), the primary motor cortex (M-I), the premotor cortex (PM), and the frontal cortices [66, 67]. Particularly, the posterior end of S-I is strongly connected to the superior parietal cortex (SPC) and, even more densely, to the anterior bank of the inferior parietal sulcus (aIPS) [68–70]. The aIPS connections themselves are also widespread and include the motor and premotor cortices, the supplementary motor cortex, S-II, PV, other areas of the posterior parietal cortex (PPC), the cingulate cortex, and the extrastriate visual cortex [67]. Although most of these connections are stronger in the outward direction than in the inward direction, anatomical evidence shows that many connections between S-I and other brain areas are bidirectional and allow an influence of S-I activity not only on other parts of the brain but also on the reverse direction [71–75].

S-I and S-II are typically activated when people observe another person receiving tactile stimulation [61, 63–65, 76]. The S-I activation is topographic, and the activation of single-finger receptive areas in S-I can be triggered purely by observing touches to different fingers [64, 77]. The degree of S-I activity during touch observation seems to be particularly strong in vision-touch synaesthetes who actually feel touch on their own body when they merely observe touch to another person's body [62]. Somatosensory cortices also respond to observing actions [51, 65, 78] and to observing haptic explorations [79] not only in humans but also in monkeys [80].

S-I is composed of altogether four subunits, three of which are mainly responsible for tactile perception (area 3b, area 1, and area 2). Whereas activation of area 1 and area 2 during touch observation is established ([63, 64, 76], but see [81]), there has been a long debate about the social response properties of area 3b, which is the homologue of S-I in other mammals [82]. Whereas some studies reported the activation of area 3b during the observation of touch [76], most studies found this area to be silent during touch observation [63, 64]. A recent study shed light on this issue. Kuehn et al. invited 16 healthy participants to a series of fMRI measurements using a 7-Tesla MRI system. Participants viewed individual touches to four fingers (index finger, middle finger, ring finger, and small finger) or received physical touches to the same four fingers in a separate scanning session [77]. Weak but fine-grained finger maps in contralateral area 3b were activated both when participants physically perceived touches at their own fingers and when they merely observed touches at another person's hand. This effect was robust across viewing perspectives but did not occur when the observed hand was not touched. The tactile-driven finger maps and the visually driven finger maps in fact overlapped in area 3b in most participants. For the first time, this study provides empirical evidence that area 3b has mirror-like response properties and that plasticity mechanisms mediated by this area should in principle be influenced by vision of touch.

Also, a number of behavioral studies showed an influence of viewing the body on somatosensory processing. Taylor-Clarke et al. showed that perceived distances between objects touching the skin are altered when participants looked at a distorted version of their body [83, 84]. Because this perceptual shift was induced by viewing the body, not by viewing the object touching the body, the effect was assumed to be driven by visual body perception. The ability to spatially discriminate two small needles applied to the skin surface ([85] but see [86]) and the ability to judge the spatial orientation of gratings touching the skin [84, 87, 88] also increased specifically when looking at one's own body compared to looking at an object. Finally, the ability to detect and discriminate the amplitude of electrical stimuli when presented to the skin clearly above threshold improved when viewing the body [89, 90].

An effect of visual body perception on tactile abilities, however, is not restricted to seeing one's own life body. They also seem to occur when participants look at a video image of a body [88, 91], at another person's body [63, 77, 92–94], or at a rubber hand [95, 96], although the effect is often stronger the more the viewed body part can be assigned to the observer's own body ([90, 95–97] but see [63]).

Evidence for a causal role of S-I in mediating tactile improvements when viewing the body was provided by a transcranial magnetic stimulation (TMS) study [98]. Here, repetitive TMS pulses were delivered to S-I or to S-II shortly after the body was visible but before the tactile stimulus arrived. TMS pulses applied to S-I, but not to S-II, diminished the effect of body vision on tactile abilities.

## 4. The Role of Touch in Human Cognition

S-I is known to be involved in the detection [99], perception [100], discrimination [101–103], and categorization [104] of touch. However, touch plays manifold roles in human cognition that go beyond the mere perception of object qualities [63, 105–107]. For example, tactile stimulation triggers emotions. Pleasant touch applied in a social context is assumed to build the basis for affiliative behavior, to contribute to the formation and maintenance of social bonds, and to build a means for communicating emotions [108, 109]. Tactile C fiber afferents particularly respond to pleasant, caress-like touch applied to hairy parts of the skin [109–111]. They terminate at the posterior insula and are assumed to elicit positive, rewarding emotions [112, 113]. Patients lacking C fiber afferents therefore perceive caress-like stroking as less pleasant than normal controls [111]. S-I may also play a role in processing affective aspects of touch [114], and it may aid in conveying socially elicited emotions to the perceiver [112].

Touch also influences human spatial perception. The incoming information in S-I is spatially ordered and represents the contralateral side of the human body in a mediolateral sequence. This body map in S-I offers a body-centered reference frame for sensory perception [115–120]. The body-centered reference frame is seen in some contrast to an external (spatial) reference frame mediated by the PPC (see [118] for a review) or the temporoparietal junction (TPJ) [121]. The body-centered reference frame may convey more self-centered information to the perceiver because information about the body as stored in S-I is assumed to be little influenced by spatial variables such as body posture [116–118, 122, 123], whereas information about the body that is stored in the PPC changes more dynamically with spatial variables (for reviews see [118, 124]).

Touch may also provide structural information about the body and its parts [125, 126]. In one experiment, participants were better in a tactile task when different tactile stimuli touched the same body part, compared to when they touched different (but adjacent) body parts [127]. Tactile processing in S-I may therefore take anatomical borders between body parts into account (see also [2]). Beauchamp et al. used multivariate pattern analyses (MVPA) to ask which aspects of body part-specific tactile processing are stored in S-I and which are stored in S-II [128]. They showed that touch applied to digits of one hand can be decoded on the basis of activity pattern in S-I, whereas gross anatomical distinctions are better decoded in S-II. Also, deafferented patients, who are deprived of somatosensory and proprioceptive input, have particular difficulties to distinctly control body parts that are nearby [129]. Finally, anaesthetizing a body part leads to an enlargement of the cortical area in S-I representing this body part [130], which presumably leads to the illusory feeling that this part of the body is larger than it actually is [131].

Touch also influences action and motor control. For example, when deprived of vision, humans have problems maintaining a stable body position. When allowed to touch an object, this supports balance, helps to control body sway [132–135], and prevents recovery falls [136]. Other examples of how tactile input influences action are haptic exploration [137, 138] or precision grips [126, 139].

## 5. The Influence of Visual Body Perception on Somatosensory Plasticity

Above, we have introduced basic mechanisms of somatosensory plasticity and learning (Section 2), discussed possible input pathways and experimental conditions that trigger nonafferent (visually driven) activations of human somatosensory cortices (Section 3), and provided an overview over the role of touch in human cognition (Section 4). Next, we will combine these research streams to target the final question, that is, “How do socially-induced ‘resonance' responses in the somatosensory system influence tactile plasticity?”

Hebbian tactile plasticity in S-I is mediated by NMDA receptors (see Section 2) that mostly reside in superficial cortical layers of the coactivated receptive field [140, 141]. So far, it is not clear whether visual signals that reach human S-I during touch observation are integrated into deeper or more superficial cortical layers in S-I. This question is relevant, because only if signals integrated into superficial cortical layers and activated similar neurons, an influence of vision of touch on S-I-mediated Hebbian learning would be expected. To target this question, Kuehn et al. [11] used the established coactivation protocol as introduced above (see Section 2) to induce S-I-mediated Hebbian plasticity in three groups of healthy participants by applying weak tactile stimulation to the tip of the index finger for the duration of three hours. Whereas one group only received tactile stimulation, two other groups were additionally presented with temporally congruent visual signals during the learning phase. One group observed object-to-hand touch; the other group observed object-to-object touch. Whereas all three groups but not the control group showed the expected tactile learning effect as measured by decreased tactile spatial discrimination thresholds after the stimulation compared to before the stimulation, there were no significant learning differences between the tactile and the two visual groups. The additional visual inputs therefore did not influence tactile plasticity to a measurable (i.e., significant) extent. Whereas different reasons can explain this finding, for example, the specific training protocol used, or different cell types that were activated by vision of touch compared to touch [142], one possibility is that visual signals integrate into deeper cortical layers in S-I. Because Hebbian learning takes place primarily in superficial cortical layers as outlined above, this would explain weak or absent effects of touch observation on Hebbian-mediated plasticity in S-I.

GABAergic inhibitory interactions are an important driving force of S-I-mediated tactile plasticity (see Section 2). Inhibitory interactions in S-I are classically characterized by measuring the relative shrinkage of index- and middle-finger receptive areas in S-I when both are activated simultaneously, compared to when they are activated alone [143, 144]. Kuehn et al. [64] replicated this effect using 7-Tesla fMRI and additionally showed that such inhibitory interactions between index-finger and middle-finger receptive areas in S-I also occur when touch to the fingers is only observed but not physically perceived. Also, a prior study has indicated an influence of vision of a body part on inhibitory interactions in somatosensory cortex during physical touch perception [87], and there is evidence that vision triggers particularly the activation of interneurons in S-I [142]. Positive correlations between levels of GABA in primary brain regions and sensory discriminative abilities stress the importance of GABA in increasing the perceived contrast of sensory percepts [43]. Weakened cortical inhibition is also a main contributor to age-related changes in somatosensation [40]. Suppressive interactions in S-I triggered by touch observation may therefore sharpen S-I receptive fields even without any afferent tactile input [64].

A single-neuron recording study in monkeys showed that there are not only (mirror) neurons that respond positively (i.e., with an increase in firing rates) to action observation but also neurons which respond negatively (i.e., with a decrease in their firing rates [145]). Although this study recorded mirror neurons in the vPM during action observation and not neurons in the somatosensory system during action or touch observation, this finding indicates that neuronal resonance responses can in principle also be inhibitory. And indeed, BOLD signals recorded in S-I during touch observation were mostly negative for the observation of noncongruent finger touches [77]. In line with this, viewing the body typically increases tactile detection thresholds [89, 90].

To study the influence of environmental conditions on somatosensory plasticity, rats were in one experimental series either reared in groups of 12 rats in spacious cages that offered multiple possibilities for object manipulation and social interaction or they were reared alone in small cages that offered fewer possibilities for object manipulation and social interaction. Rats that were reared in groups and in spacious cages showed an expansion of the forepaw maps in S-I compared to those who were reared alone and in an impoverished environment [146]. This effect occurred both for young and older rats [147]. However, it cannot be derived from these studies whether the effects were driven by increased rates of object manipulation (i.e., sensorimotor experience) and/or the presence of social interaction partners (i.e., social touch). Dissociating both influences on somatosensory plasticity would be an important goal for future research.

Rubber hands are an often-used tool to study the influence of visuotactile stimulation on bodily awareness. Press et al. [148] used a similar paradigm to study the influence of vision on somatosensory plasticity. They applied touches to a rubber hand or to a rubber object when participants perceived either synchronous or asynchronous touches at their own hand. After the bimodal (synchronous or asynchronous) training, ERPs over somatosensory cortex were measured in response to unimodal tactile stimulation to the hand. The temporal contingency of visuotactile stimulation delivered during the training phase influenced the ERPs in response to pure tactile stimulation: those participants who trained with synchronous visuotactile stimulation showed an enhanced somatosensory N140 component compared to those who trained with asynchronous visuotactile stimulation. The N140 component is assumed to be elicited in S-II, which contains bilateral receptive fields. This may explain why the effect was not side specific but occurred for both hands. The enhanced N140 after the learning was found both after participants observed touch to a hand and after they observed touch to an object. Classical mirror mechanisms were likely not at play but perhaps bottom-up effects mediated by multisensory integration.

As introduced above (Section 4), touch plays a significant role in emotion perception. Disrupting S-I activity impairs the ability to recognize emotional facial expressions in peers [149–151], and S-I plays a role in recognizing emotional voices [152]. Somatosensory plasticity may therefore also influence emotion perception, such as those elicited by social stimuli. To study this, Friedrich et al. [153] conducted a training study on children with autism spectrum disorder (ASD). For the duration of 6 to 10 weeks, children were trained to either increase mu power (group 1) as measured with EEG over somatosensory cortex or decrease mu power (group 2) when performing a social interaction video game. Suppression of mu power is assumed to reflect neuronal resonance responses in the somatosensory cortex. When comparing pretraining with posttraining mu suppression during an independent task that was not used during training and where children observed emotional facial expressions, only group 2 showed more mu suppression after the training compared to before the training and also showed more mu suppression than group 1 after the training. It is worth noting that other outcome measures did not differ between groups. Training the responsivity of the somatosensory cortex during social perception may therefore enhance empathic responses towards emotional conspecifics also in situations that were not part of the training data set. Further work will have to confirm this finding.

As outlined above (see Section 4), touch contributes to a body-centered reference frame. S-I activity during touch observation may therefore trigger the ability to “put yourself into the shoes of others” [78]. The positive correlation between S-I activity during touch observation and perspective taking abilities as assessed by questionnaires ([154], see similar results in [78]) may be interpreted in this direction. Physical touch perception, on the other hand, could prevent undertaking such a shift in reference frames. This is indicated by a study of Palluel et al. [155]. Here, a virtual reality setup was chosen that allowed showing participants their own back in front. They saw their own back being stroked by brushes, either synchronously or asynchronously to the stroking they felt on their real back. This situation typically triggers participants to feel that the virtual back is their own back, which induces a strong visuotactile interference effect (see also [156]). When participants were stimulated by vibration stimuli on their leg, however, they did not feel the illusion anymore, and they also did not show the visuotactile interference effect [155]. One may argue that the perceived leg vibration caused an activation of their own body-centered reference frame, which prevented them from taking over the other person's reference frame. To the best of our knowledge, so far, no study has specifically studied the effect of tactile training on social perspective taking. The above-outlined studies would indicate a reverse relationship.

Touch observation has also been used to study clinical populations, such as limb amputees, and extinction patients. Hand amputees are an often used model system to study somatosensory plasticity in humans. As outlined above (see Section 2), in spite of an absent hand, limb amputees show an astonishingly intact and only slightly shifted representation of the missing hand in the sensorimotor cortex. It has been argued that both the degree of distortion [29] and the degree of preservation [31] of somatotopic maps in limb amputees contribute to the perception of phantom limb pain. To induce activation and/or to modify the representation of the S-I missing-hand territory in amputees therefore seems to be a goal worth pursuing. Again, the rubber hand illusion may be a suitable tool. Ehrsson et al. [157] showed that observing a rubber hand that is touched synchronously to the stump evokes the illusion in upper limb amputees that the observed hand is their own hand, even though in fact their hand is missing. This effect was present using different psychophysical markers and was also seen in self-report questionnaires. Also, Goller et al. [158] showed that when limb amputees observe another person being touched at different body sites, some of them start feeling touch on their own phantom limb (see also [159]). This did not only occur in patients who frequently experienced phantom limb sensations but also in patients who reported experiencing phantom limb sensations only occasionally, or not at all. Similar to the mirror-box illusion where the moving intact hand creates the illusion of a moving missing hand, also the rubber hand illusion may serve as a therapeutic tool for influencing somatosensory plasticity in limb amputees. However, congenital limb amputees do not show S-I activity when observing another person in pain [160], which indicates possible functional differences between S-I responsivity to touch and pain in limb amputees.

Extinction impairs the ability to perceive multiple stimuli of the same type simultaneously and occurs usually after damage to the contralateral hemisphere. One study investigated whether also the visual presentation of a rubber hand can cause tactile extinction in patients with right brain damage [161]. In patients with left tactile extinction, a visual stimulus was presented near a right rubber hand and near the real right hand. The rubber hand condition induced visuotactile extinction similar to the real hand, indicating that tactile extinction is not specific for perceiving one's own body, but it can also be induced by observing another person's body.

Finally, as outlined above (see Section 3), besides mirroring observed touches, S-I also responds to the observation of human movements, and S-I influences different aspects of human action and motor control (see Section 4). In this last paragraph, we therefore concern with the interaction between action observation, motor resonance, and somatosensory plasticity. TMS is an often-used tool to induce or modulate cortical plasticity. Avenanti et al. [162] investigated the specific influence of virtual lesions in S-I as induced by repetitive TMS (rTMS) over S-I on motor-evoked potentials (MEPs) measured at the hand during observed hand movements. The authors found rTMS to specifically disrupt the ability to resonate with extreme joint-stretching finger movements that induced, by subjective report, strong tactile/proprioceptive sensations during observation. In a different study, TMS pulses delivered over S-I disrupted the ability to correctly judge the weight of a box lifted by a hand but not the ability to correctly judge the weight of a bouncing ball [163]. A contribution of S-I to proprioceptively driven weight judgments has also been indicated by a patient study. Here, deafferented patients were shown to be impaired in their ability to correctly estimate the weight of a box lifted by a person [164]. On the other hand, TMS-adaptation (TMS-A) over S-I can be used for behavioral enhancement [165]. Jacquet and Avenanti showed that TMS-A over S-I leads to a reduction in reaction times when participants were asked to recognize the goal (but not the movement) of an observed hand movement. Somatosensory plasticity can therefore potentially be used to enhance empathic abilities during action observation. There also seems to be the potential to use action observation to induce somatosensory plasticity.

## 6. Summary

Converging evidences from human and monkey research support the notion that S-I is not only involved in the detection, perception, discrimination, and categorization of touch but also linked to more complex cognitive and emotional functions. More recent work even proposes S-I as a reference frame for social “resonance” that involves those subareas formally assumed to only respond to “real” physical tactile inputs arising from the thalamus [77]. This raises the fundamental question of whether social tactile cues may induce or boost tactile processing, perception, and even plasticity [11] and whether this may offer new treatment options, for instance, in phantom limb pain, stroke rehabilitation, or even social distortions. Future research is needed to understand the functional role of cortical social “resonance” in primary and further downstream sensory regions and their specific contribution to perception and plasticity.
